# The Associations between Results in Different Domains of Cognitive and Psychomotor Abilities Measured in Medical Students †

**DOI:** 10.3390/brainsci13020185

**Published:** 2023-01-22

**Authors:** Ivana Pavlinac Dodig, Aisha Qazzafi, Linda Lusic Kalcina, Sijana Demirovic, Renata Pecotic, Maja Valic, Zoran Dogas

**Affiliations:** 1 Department of Neuroscience and Split Sleep Medicine Center, University of Split School of Medicine, 21000 Split, Croatia; 2 Department of Neuroscience, University of Split School of Medicine, 21000 Split, Croatia

**Keywords:** cognition, psychomotor performance, medical students, intelligence test

## Abstract

We aimed to investigate the associations between intelligence quotient test scores obtained using the Raven’s Advanced Progressive Matrices (APM) and psychomotor testing using the Complex Reactionmeter Drenovac (CRD) test battery, while taking into account previous theoretical approaches recognizing intelligent behavior as the cumulative result of a general biological speed factor reflected in the reaction time for perceptual detections and motor decisions. A total of 224 medical students at the University of Split School of Medicine were recruited. Their IQ scores were assessed using Raven’s APM, while the computerized tests of CRD-series were used for testing the reaction time of perception to visual stimulus (CRD311), psychomotor limbs coordination task (CRD411), and solving simple arithmetic operations (CRD11). The total test-solving (TTST) and the minimum single-task-solving (MinT) times were analyzed. On the CRD11 test, task-solving times were shorter in students with higher APM scores (r = −0.48 for TTST and r = −0.44 for MinT; *p* < 0.001 for both). Negative associations between task-solving times and APM scores were reported on CRD311 (r = −0.30 for TTST and r = −0.33 for MinT, *p* < 0.001 for both). Negative associations between task-solving times in CRD411 and APM scores (r = −0.40 for TTST and r = −0.30 for MinT, *p* < 0.001 for both) were found. Faster reaction time in psychomotor limbs coordination tasks, the reaction time of perception to visual stimulus, and the reaction time of solving simple arithmetic operations were associated with a higher APM score in medical students, indicating the importance of mental speed in intelligence test performance. However, executive system functions, such as attention, planning, and goal weighting, might also impact cognitive abilities and should be considered in future research.

## 1. Introduction

Cognitive actions, such as identifying the problem, processing information, creating a plan for the next step, carrying out this plan, and then evaluating the results, are necessary for medical students, especially in terms of medical decision making and problem solving [1]. More specifically, cognitive functions, such as executive functioning and working memory, are crucial in reasoning, the ability to problem-solve and acquiring new skills and knowledge [2]. Processing speed, as a factor that represents one’s cognitive performance in a specific amount of time and, therefore, the ability to process information quickly, is also strongly correlated with the ability to reason and with higher-order cognition [2,3,4,5,6,7].

Intelligence, the ability to acquire and apply knowledge and skills, integrates cognitive functions, such as perception, attention, memory, language, or planning [8,9]. It has been demonstrated that intelligence is a major predictor of success in education and occupations [10]. There is a positive association of educational attainment with intelligence test scores [11], and it has been shown that for each year of education, there is an increase in intelligence test scores [12]. Furthermore, education does have domain-specific effects, such as improvement in memory and reasoning ability [13]. However, education develops only certain, specific intellectual abilities, rather than general intelligence. Although the efficiency of cognitive operations may not be improved with education, the development of specific cognitive domains, such as memory, can have an extensive impact on cognitive development [13,14].

It has been shown that when students performed well in one subject, they would also perform well in other subjects. Even when the test results were discrepant, such as in scientific reasoning and standardized ability test, the correlation between the test results was improved by changing the format of the test presentation [7,15,16]. Such a positive correlation between performances on different tests indicates that there is an underlying factor conducting this success [17], known as the general intelligence factor. General intelligence refers to having reasoning ability and behavioral flexibility, and it is a good predictor for several components of life outcomes, including academic achievement, the probability of success in professional careers, occupational attainment, job performance, social mobility, and even health and survival [9,18,19].

Previous research has identified a positive correlation between different domains of cognitive performance and intelligence tests, implying that subjects who excel on one test would also excel on other cognitive tests. Especially strong associations with IQ were found for processing speed, working memory, and visual and verbal learning [20,21]. 

The theoretical background of the current research is found in an extensively elaborated relationship between psychometric intelligence and reaction times (RTs). The former association has been thoroughly described in three well-known elementary cognitive tasks (Hick simple and choice reaction time task, Sternberg’s short-term memory scanning, and Posner’s letter matching), with short reaction times (RTs) being associated with higher intelligence [16]. Such association has been explained with a proposal for a higher speed of information processing, reflecting a physiological difference. It should be underlined that theories contradicting these findings have also been published, suggesting that brain structural properties may influence both mental speed and general intelligence, giving rise to reported associations [20]. The direction of the association of reaction time and intelligence is therefore still inconclusive.

A battery test, Complex Reactionmeter Drenovac (CRD-series), is a computer-generated chronometric instrument measuring perception, memory, psychomotor reaction, and thinking as dynamic functions of the central nervous system and attention, as well as functional disturbances [22,23,24]. Fluid intelligence is usually assessed by nonverbal tests that require inductive reasoning and spatial visualization, and one of the most common tests used is Raven’s Advanced Progressive Matrices (APM) [25]. However, the association between intelligence tests performed through the Raven’s APM test and cognitive and psychomotor testing performed using the CRD-series has not yet been reported.

Using the CRD-series to test cognitive and psychomotor abilities provides additional information on performance stability during the testing process, represented through ballast measures. Investigating reaction time stability as a unique and accurate indicator of cognitive and psychomotor stability [26,27,28] provides complementary data to the existing literature on the association of cognitive abilities. Additionally, the use of the CRD-series enables the assessment of convergent thinking. Differentiation of divergent and convergent thinking was postulated in Guilford’s models, suggesting that both aspects are required in problem solving [29]. The development of and selection of ideas in working towards the optimal solution to a problem reflects convergent thinking [30]. It has been investigated and confirmed that mathematics performance mainly relies on convergent thinking, and theories supporting a relationship with mathematics performance suggest that convergent thinking aids in the evaluation of ideas and contributes to the finding of a good solution to a problem [31].

Furthermore, employment of medical students as a specific population in this research yields specific added value to the research. The lack of studies investigating reaction times among medical students has been previously emphasized [32]. Medical students are exposed to quite demanding schedules and high academic demands during their study [33,34], consequently leading to an increased stress load that might have detrimental effects on various cognitive abilities and reaction time [35]. To our knowledge, this is the first study reporting different aspects of reaction time performance, as well as the variability in reaction time during the test. One of the investigated measures of reaction time enabled the assessment of the reaction time of both the upper and lower extremities, rarely reported in this group of students. It is well known that shift work, to which doctors are often exposed, might decrease their reaction time [36,37]. Taking this into account, a comprehensive and thorough understanding of the reaction time performances of medical students is mandatory to enable an assessment of a longitudinal variability in various reaction time performances of future residents and doctors.

Thus, this study aims to investigate if there is an association between IQ test scores assessed with the Raven’s APM and cognitive and psychomotor testing through the CRD-series test battery in medical students. The hypothesis of the current research may be elaborated based on the mental speed theories of intelligence, suggesting that intelligent behavior may be seen as the cumulative result of a general biological speed factor reflected in the time required to make basic perceptual detections and motor decisions [6]. We hypothesize that a faster and more accurate performance on reaction time in the psychomotor limbs coordination task, the reaction time of perception to visual stimulus, and the reaction time of solving simple arithmetic operations, assessed by an electronic psycho-diagnostic test battery, will be associated with a higher score achieved on Raven’s APM in medical students.

## 2. Materials and Methods

The Biomedical Research Ethics Committee at the University of Split School of Medicine approved the protocol of this study (Approval number 33-1/06). All procedures were done in accordance with the ethical standards from the 1964 Helsinki declaration and its later amendments. All participants signed informed consent prior to the study protocol.

### 2.1. Participants

The participants involved in this study were second-year medical students at the University of Split School of Medicine (USSM) studying in the English and Croatian programs. From 2017 to 2019, a total of 224 subjects were recruited for participation in the study. Exclusion criteria were refusing to participate in this study and not being present at the examination. The participants were not asked to prepare in advance for any of the tests. All the tests were performed in the morning hours from 8 am to 11 am in a quiet laboratory room at the USSM.

### 2.2. Intelligence Testing

The intelligence of the students was measured through a multiple-choice, pencil-and-paper test version of the nonverbal test Raven’s APM. Students had 45 min to complete as many of the 36 items of the abstract reasoning test as possible. Each item required the student to identify the missing element that would complete the 3 × 3 matrix of patterns presented. There was increasing difficulty with each test item, and the student was given a choice out of eight possible answers and a “Do not know” option. The number of items answered correctly out of 36 was the score used for measuring performance on this test. APM provides a standardized cognitive ability test, primarily designed to measure high-level observation skills, clear thinking ability, and intellectual capacity as a non-verbal estimate of abstract reasoning or fluid intelligence [38]. It has been suggested by Spearman that Ravens matrices require both the eduction of correlates (predicting the configuration of the elements given the relation), as well as the eduction of relations (abstracting a relation from the configuration of concrete elements) [25].

### 2.3. Cognitive and Psychomotor Performance Testing

**Figure 1:**
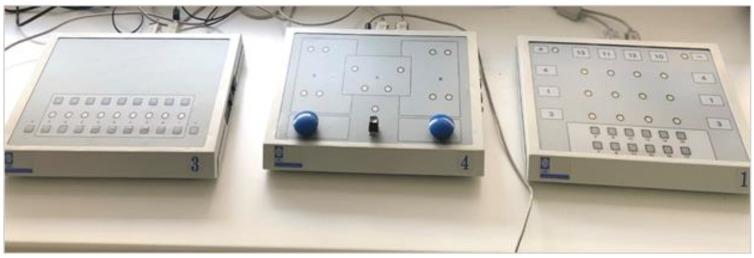
Complex Reactionmeter Drenovac (CRD)-series. In this research, test CRD311 assessing discrimination of simple visual stimulus was solved on the left panel marked with number 3, test CRD411 assessing complex psychomotor coordination was solved on the middle panel marked with number 4, and test CRD11 assessing convergent thinking through solving simple mathematical operations was solved on the right panel marked with number 1. The light stimulus in all tests was presented in small circles on each panel.

**Figure 2:**
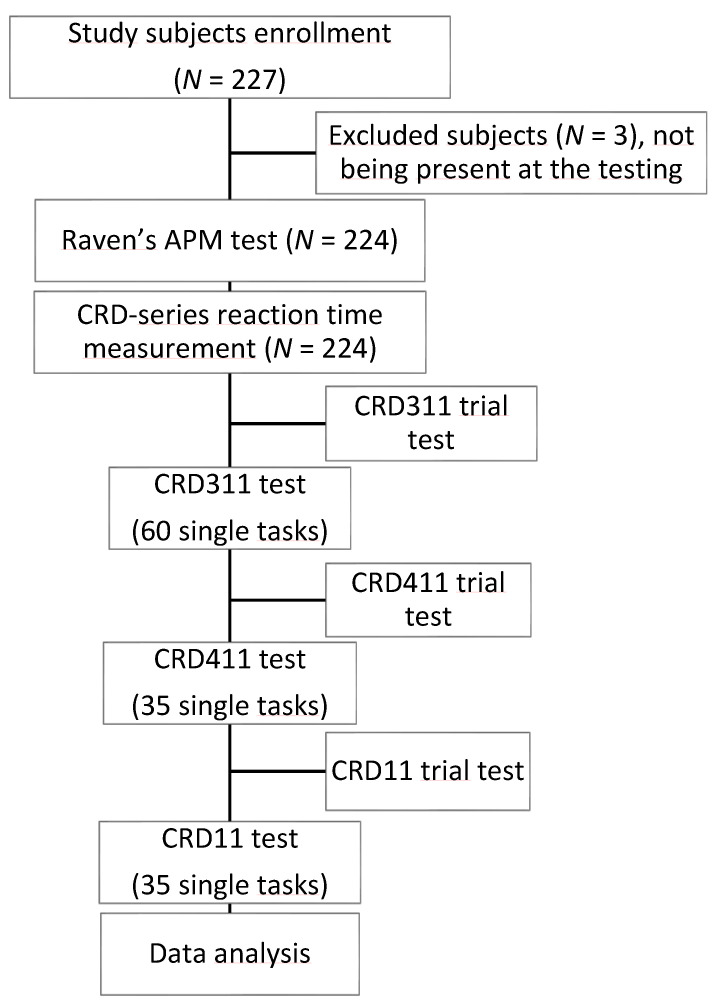
Study flow chart. A total of 224 medical students were tested with Raven’s APM and three tests (CRD311, CRD411, and CRD11) of the CRD-series test battery. APM = Advanced Progressive Matrices, CRD = Complex Reactionmeter Drenovac.

The computerized tests of the CRD-series shown in Figure 1 were used for testing the cognitive and psychomotor performance of the participants. The time needed to process information in this chronometric test represents the psychomotor function of the reaction time. For this investigation, three different tests from the CRD-series were used in the same order, from the simplest to the most difficult test: the light signal discrimination test, measuring the reaction time of perception to a visual stimulus (CRD311); the test of operative thinking, measuring reaction time in a psychomotor coordination task of participants’ upper and lower limbs (CRD411); and the test of convergent thinking, through solving simple arithmetic operations of addition and subtraction (CRD11), as shown in Figure 2. The CRD311 test included 60 tasks in a random sequence, appearing on a panel in nine circles arranged in one row (Figure 1). There was only one light presented at the time, and the subjects were instructed to press the corresponding square button below that light as quickly as possible when the light appeared. The square buttons had to be pressed using the index finger of the dominant hand. Once the correct button was pressed, the light would turn off and a new light would appear on the panel. The participant had to continue to press the appropriate button under the light until the whole sequence of 60 lights was displayed.

As for the CRD411 test, there was a panel where up to four lights could appear at the same time, arranged with two in the top row and two in the bottom row (Figure 1). Following the light appearance, participants would need to press the button with their corresponding hand, depending on whether the light appeared on the left or right side in the top row. The two lights of the bottom row, whether left or right, required that the individual push the pedal on the floor with their corresponding foot. It was possible for one, two, or three lights to appear in this 35-item test, which could involve the student pressing both hands or both feet, or one hand or one foot, or all possible combinations of hands and feet if all three lights appeared. By pressing the appropriate buttons and/or pedals, the task would be correctly solved, and a new light or combination of up to three lights would be turned on. The next task, represented by a new light or combination of lights, would begin once the previous task was answered correctly, and if more than one light was displayed, the corresponding buttons or pedals had to be pressed simultaneously for the participant to answer the task correctly.

In the CRD11 test, the subjects had to perform simple arithmetic operations of summation or subtraction. In the CRD device panel, there were 12 lights centrally in small circles, organized in four columns and three rows (Figure 1). Above each column, as well as to the right or left of each row, was a number that had to be used for summation or subtraction. Once the light appeared, it would indicate which numbers had to be used in the mathematical operation. Thus, one light presented centrally would indicate the numbers just above it and to the right/left of it to be used. An additional light next to a (+) or with a (−) sign would appear in the left/right corner of the panel to indicate whether the task needed summation or subtraction. The greatest number on the panel was 13, and there were 35 tasks to solve for the test to end. Subjects were instructed to press the correct answer by pressing the result of the sum or the subtraction of the indicated numbers (from 6 to 17), presented in the bottom row. The next task appeared once the student correctly answered the previous task. Before commencing each CRD test, the students were allowed a trial test to familiarize themselves with the dynamics of the test to prevent overwhelming the participant. Each student had the same variation of each test, so there were no differences in complexity, and they were all required to complete each test as quickly and accurately as possible. The study flow chart is shown in Figure 2.

In our protocol, there were no color-based responses, as all the light signals used in the test were presented in the same color. Furthermore, all the participants had no severe visual impairments, and myopia/hyperopia was appropriately corrected.

### 2.4. Data Collection and Statistical Analysis

Six parameters of each test of the CRD-series were recorded and analyzed: total test-solving time (TTST), minimum single-task-solving time (MinT), median single-task-solving time (MedT), start ballast (ST), end ballast (EB), and total ballast (TB). The indicators of speed, accuracy, and mental endurance were TTST, MinT, and MedT, respectively. The ballasts were descriptors of stability and showed the time that was wasted and not spent on solving the tests. It was calculated as the sum of the differences between the time spent on each individual task (Ti) and a minimum single-task-solving time. Start ballast (SB) represented the wasted time during the first half of the test, the end ballast (EB) the wasted time during the second half of the test, and the total ballast (TB) was the sum of SB and EB, i.e., the total wasted time during the whole test.

For statistical analysis, MedCalc for Windows version 11.5.1.0 (MedCalc Software, Mariakerke, Belgium) was used. The distribution of data was assessed for normality with the use of the Shapiro–Wilk or Kolmogorov–Smirnov test. In cases with asymmetric data distribution assessed with a significant Shapiro–Wilk and Kolmogorov–Smirnov test, a parametric analysis was performed due to a large sample size, as well as following inspection of Q-Q plots and the skewness or kurtosis of the asymmetrically distributed data. Categorical data were presented as absolute and relative frequencies, while continuous variables were presented as mean ± standard deviation, and the age of subjects was shown as median (minimum, maximum). The association between the performances on the CRD-series tests and Raven’s APM intelligence test was tested using Pearson’s correlation coefficient. The contribution of the CRD-series test results to Raven’s APM test was analyzed using multiple linear regression. The level of statistical significance was set at *p* < 0.05.

The data that support the findings of this study are available from the corresponding author, upon reasonable request.

## 3. Results

There was a total of 224 medical students involved in this study, of which 72 (32.1%) were men and 152 (67.9%) were women. The median age was 21 years (Table 1).

### 3.1. Performance on the Raven’s APM and CRD-Series of Study Participants

**Figure 3:**
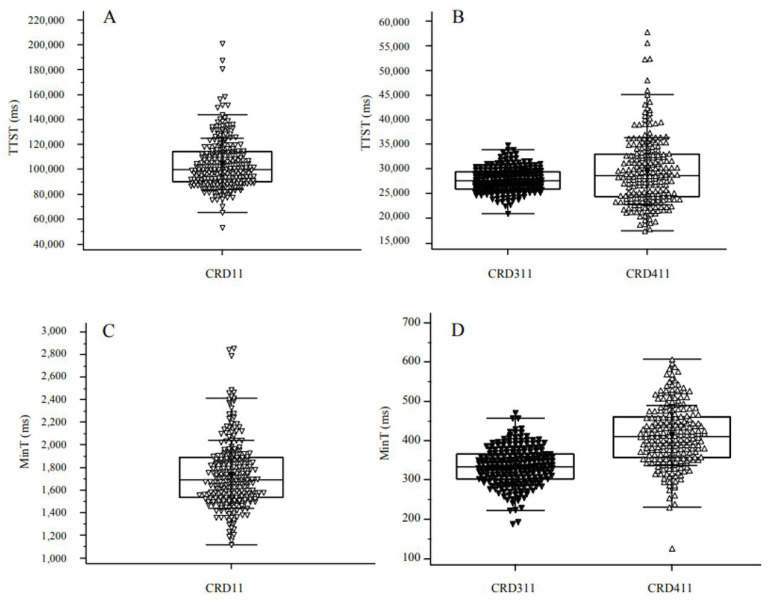
Total test-solving time (**A**,**B**) and minimum single-task-solving time (**C**,**D**) on test CRD11 (**A**,**C**) and tests CRD311 and CRD411 (**B**,**D**). Each panel shows overlying individual data points, with each point representing results of a single subject. CRD = Complex Reactionmeter Drenovac, TTST = total test-solving time, MinT = minimum single-task-solving time.

The medical students in this study had a mean Raven’s APM score of 27.12 (Table 1). Performance on the CRD11, CRD311, and CRD411 tests of the CRD-series is summarized in Table 1 and shown in detail in Figure 3.

### 3.2. Association between Performance on Raven’s APM Intelligence Test and CRD-Series Tests

**Figure 4:**
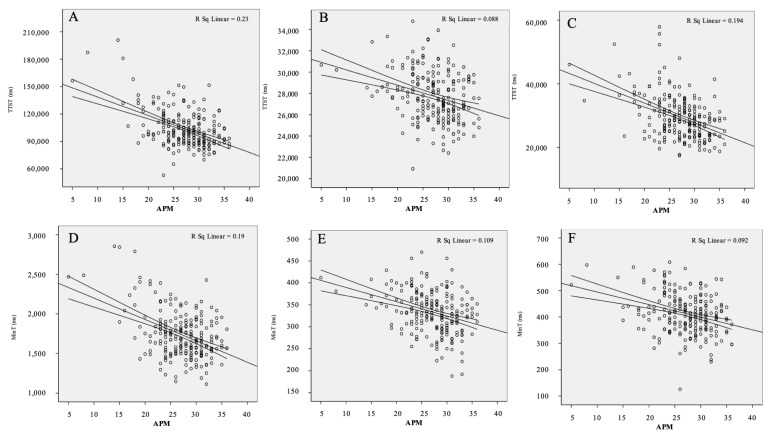
The correlations between results on CRD-series and APM scores. On upper panels ((**A**) for test CRD11, (**B**) for test CRD311, and (**C**) for test CRD411) are shown correlations between total test-solving times with APM scores, while on lower panels ((**D**) for test CRD11, (**E**) for test CRD311, and (**F**) for test CRD411) are shown correlations between minimum single-task-solving time and APM score. There were significant negative correlations between APM score and results on three tests of the CRD-series, where longer TTST and MinT on each CRD-series test was correlated with lower APM score. APM = Advanced Progressive Matrices, CRD = Complex Reactionmeter Drenovac, TTST = total test-solving time, MinT = minimum single-task-solving time.

The negative association between the performance on Raven’s APM intelligence test and the majority of the CRD-series tests is reported in Table 2 and Table S1, and shown in Figure 4. On the reaction time of the solving simple arithmetic operations test, task solving times were shorter in students with a higher APM score (r = −0.48 for TTST and r = −0.44 for MinT; *p* < 0.001 for both tests). A similar negative correlation was found for the ballast times and APM scores (r = −0.41, −0.24, −0.36; for SB, EB, and TB, respectively, all with *p* < 0.001).

On the reaction time of the perception to visual stimulus test, there are shorter task-solving times in correlation with a greater APM score (r = −0.30 for TTST and r = −0.33 for MinT, *p* < 0.001 for both variables). However, there was no significant association between the ballast times and the APM scores of the medical students.

As for the reaction time in the psychomotor limbs coordination task test, there was a negative correlation between task-solving times and APM scores, and with a higher APM score, task-solving times were shorter (r = −0.40 for TTST and r = −0.30 for MinT, *p* < 0.001 for both). Ballast times also demonstrated a negative correlation with APM scores (r = −0.41, −0.34, and −0.40 for SB, EB, and TB, respectively, all with *p* < 0.001).

### 3.3. The Contribution of Age, Gender, and CRD-Series Test Results to APM Score

A multiple linear regression was performed to analyze the contribution of age, gender, and CRD-series test results to the APM score (Table 3). Out of the analyzed variables, TTST on tests assessing the reaction time of solving simple arithmetic operations and the psychomotor limbs coordination task (β = −0.182, *p* = 0.009, and β = −0.215, *p* = 0.002, respectively), as well as MinT on a test assessing reaction time of perception to a visual stimulus (β = −0.158, *p* = 0.023), significantly contributed to the APM score.

## 4. Discussion

This study demonstrated that faster reaction time on different psychomotor reaction tests performed with the use of a psychodiagnostic test battery was associated with higher Raven’s APM scores. On the light signal position discrimination tests, students who were faster at accurately discriminating light signals had higher Raven’s APM scores, compared to students reacting slower. Likewise, students faster at accurately solving simple arithmetic operations, as well as those reacting faster on psychomotor coordination tasks of participants’ upper and lower limbs, had higher APM scores, while also having better stability of performance. This implies that medical students reacting faster when assessed with a computerized psychodiagnostic test battery had an overall greater APM score.

Our findings are in accordance with mental speed theories of intelligence, suggesting that intelligent behavior may be seen as the cumulative result of a general biological speed factor reflected in the time required to make basic perceptual detections and motor decisions [6,39].

Given that the speed of perception is a subdomain of cognitive function [40], a faster response at detecting the light stimulus might have led to a higher APM score. Similar results were found in a study exploring the relationship between intelligence and visual recognition memory in adolescents, where higher scores on intelligence tests were associated with a faster time in answering the visual recognition memory test [41]. Furthermore, it was revealed that children performing significantly better on a variety of cognitive and psychomotor tests had greater intelligence, with the strongest relationship on the tasks involving the visuospatial processing domain [42]. Thus, one might presume that the subjects with better perceptive abilities, who are more likely to process information at a faster rate, might have higher intelligence.

Since convergent thinking encompasses the use of logic, accuracy, and speed, and essentially means the ability to give a correct answer to a question, it might be evaluated with the use of a test assessing the reaction time of solving simple arithmetic operations [43]. Our results revealed that a better performance on the test of convergent thinking correlates with higher intelligence. This is probably due to being able to provide correct answers quickly to these arithmetic operations, and medical students in general often already have had greater mathematical knowledge and arithmetic skills with secondary school grades for math reflecting this. In a previous study, a positive correlation was found between convergent thinking assessed through calculation [44] and intelligence estimated by measuring one-word receptive vocabulary in elderly patients with and without brain dysfunction [45]. Thus, this study revealed that a greater intelligence would be associated with better cognitive performance, including convergent thinking [45], which is also represented in our study.

As for psychomotor skills, another cognitive function, they involve an organized and well-coordinated pattern of muscular activities prompted by environmental signals [23]. Likewise, faster reaction times on the test of psychomotor performance could be explained by a greater speed of processing, visuospatial abilities, and possibly better attention, which were all associated with a greater intelligence [21,42]. A previous study showed that most of the primary cognitive domains, including attention, psychomotor speed, episodic memory, working memory, and executive functioning, correlated with each other. It was also seen that higher intelligence was associated with better performances on the psychomotor test and executive functioning test [46]. Another study demonstrated a positive correlation between intelligence testing and cognitive performance, including simple psychomotor abilities, such as finger-tapping speed [47]. This further reinforces the evidence of a greater APM score correlating with better achievement on the reaction time in the psychomotor limbs coordination task test in the present study. We emphasize that low statistical power should be taken into account in the current study, since it might have increased the likelihood of a false positive result. Thus, correlations, such as those reported in our study, obtained with small samples may be unreliable, taking into account that the small sample size may increase the likelihood of obtaining a false positive large correlation coefficient.

Even though the direction of the association of reaction time and intelligence is still inconclusive, it has been proposed that even higher associations are found with increasing informational load [7]. Such findings are often elaborated with Spearman’s concept of general intelligence, as well as with proposals suggesting that more complex tests can better assess general intelligence. The advantage of the current study on this matter, is the use of different reaction time tasks of variable complexity. The current study reported that the total test-solving times on both tests of higher complexity, assessing the reaction time of solving simple arithmetic operations and psychomotor limbs coordination, had a significant contribution to the APM score. For the less complex test assessing reaction time of perception to visual stimulus, only MinT significantly contributed to the APM score. These findings recognize the speed and accuracy of the cognitive performance, as well as the mental endurance assessed by CRD-series tests, contributing significantly to intelligence. Our results are also in accordance with the results of the previous studies, which confirmed that there is an association between different domains of cognitive performance and intelligence; thus, individuals who excel on one test would also excel on other cognitive tests [21,45,46,47].

It is convincing that the students’ academic abilities and educational background may have provided them with better cognitive abilities. However, our result of an average Raven’s APM score among medical students is in accordance with the norms for undergraduate students. More precisely, a score of 27 equates to the 50th percentile ranking for undergraduate students [48], indicating that participants of this study were representative of the student population. However, caution has to be made regarding the generalizability of our results, considering that people with high intelligence, but with physical impairments, such as symptoms related to neurological conditions or trauma consequences, cannot perform well on the tasks used in the study.

The unequal proportion of genders in our study was the consequence of a higher proportion of female students enrolled in USSM, reflecting a worldwide trend of the feminization of medicine [49]. However, we believe that these unequal gender proportions did not influence our results. It has been previously shown that men achieved better results than women on convergent thinking (CRD11) and psychomotor (CRD411) tests [23], and that men usually have better calculation skills, and they typically have better motor and visuospatial skills, while females are better in verbal abilities [50,51,52]. Still, both genders share several similarities regarding their cognitive abilities [50,51,52], which is in accordance with our results, with no gender differences in the results of the Raven’s APM tests.

This study is not without limitations. Even though we used APM, which is one of the most commonly used tests in the assessment of fluid intelligence, we did not include an assessment of other types of intelligence, and no comprehensive assessments of verbal aspects of intelligence were performed.

Among the limitations, one might question the relevance of the participants’ population, students of the Croatian and English programs of medical studies. Still, we believe that this did not influence the results, given that neither the APM nor the CRD-series tests are culture or language sensitive. Other factors, such as lifestyle and sleep habits, as well as chronotype, might have had an impact on the students’ performance, especially bearing in mind that all the tests were carried out in the morning hours from 8 am to 11 am. However, we believe that accounting for these confounders would not materially change the observed effects since the observed effects were large. Further, there was no data from other distinctive populations that enabled comparison with our data obtained on medical students. Finally, current findings should be interpreted with caution, since a small study sample might have contributed to low statistical power, resulting in inflated effects compared to a true effect. Hence, a statistically significant correlation of CRD and APM results might represent a false-positive result, since a low sample size of the current study might negatively affect the likelihood that a nominally statistically significant finding indeed reflects a true effect.

## 5. Conclusions

In conclusion, this study is the first to examine the association between Raven’s APM scores and cognitive abilities testing through the CRD-series test battery in medical students, and it indicated that faster reaction times in tasks of convergent thinking, operative thinking, and speed of perception are associated with higher APM score. Thus, we recognized the relevant role of mental speed in intelligence. It should be underlined that the role of attention in the assessment of the association of reaction time performances and intelligence has also been investigated, and it has been suggested that both goal weighting and planning have a role in intelligent behavior, as part of the executive system functions [6]. These constructs should be considered in future studies; but we emphasize that the current study aimed specifically to investigate the possible association of reaction time in psychomotor tasks of different complexity and performance on Raven’s APM intelligence test as a measure of fluid intelligence.

## Figures and Tables

**Table 1:** Demographic characteristics and performance of the Raven’s APM and CRD-series of study participants.

	Total (*N* = 224)		
Age (years)	21 (19–31)		
Croatian program, N (%)	171 (76.3)		
English program, N (%)	53 (23.7)		
Cognitive test results			
Raven’s APM score	27.12 ± 4.85		
CRD testing results	CRD11	CRD311	CRD411
TTST (s)	104.38 ± 20.54	27.81 ± 2.41	29.52 ± 6.87
MinT (s)	1.74 ± 0.30	0.34 ± 0.05	0.41 ± 0.08
SB (s)	19.93 ± 7.57	4.02 ± 1.01	4.94 ± 0.20
EB (s)	23.56 ± 9.28	3.71 ± 0.98	10.12 ± 4.02
TB (s)	43.49 ± 14.78	7.73 ± 1.84	15.06 ± 5.54
NoErr	2.92 ± 2.65	0 ± 0	8.38 ± 5.54

Age is shown as median (minimum–maximum), Raven’s APM score and CRD testing results are presented as mean ± standard deviation. APM = Advanced Progressive Matrices, CRD = Complex Reactionmeter Drenovac, TTST = total test-solving time, MinT = minimum single-task-solving time, SB = start ballast, EB = end ballast, TB = total ballast, NoErr = number of errors.

**Table 2:** Correlation between Raven’s APM scores and performance on CRD-series testing.

	CRD11		CRD311		CRD411	
	r	*p* ^1^	r	*p* ^1^	r	*p* ^1^
TTST	−0.48	<0.001	−0.30	<0.001	−0.44	<0.001
MinT	−0.44	<0.001	−0.33	<0.001	−0.30	<0.001
SB	−0.41	<0.001	0.12	0.073	−0.41	<0.001
EB	−0.24	<0.001	0.13	0.052	−0.34	<0.001
TB	−0.36	<0.001	0.14	0.036	−0.40	<0.001
NoErr	−0.08	0.233			−0.15	0.025

CRD = Complex Reactionmeter Drenovac, TTST = total test-solving time, MinT = minimum single-task-solving time, SB = start ballast, EB = end ballast, TB = total ballast, NoErr = number of errors. ^1^ *p* values were calculated with the use of Pearson’s correlation coefficient.

**Table 3:** Regression analysis of APM score based on the multiple linear regression model including total test-solving time (TTST) and minimum single-task-solving time (MinT) on CRD11, CRD311, and CRD411 tests, age and gender of medical students assessed in the study.

	R^2^	*p* *	β	t	*p* *
Age	0.279	<0.001	−0.132	−1.901	0.059
Gender			0.089	1.278	0.203
CRD11					
TTST			−0.182	−2.650	0.009
MinT			−0.037	−0.531	0.596
CRD311					
TTST			0.052	0.746	0.457
MinT			−0.158	−2.297	0.023
CRD411					
TTST			−0.215	−3.147	0.002
MinT			<0.001	0.007	0.994

CRD = Complex Reactionmeter Drenovac, TTST = total test-solving time, MinT = minimum single-task-solving time *p* * values were calculated with the use of multiple linear regression.

## Data Availability

The datasets generated during and/or analyzed during the current study are available from the corresponding author on reasonable request.

## Supplementary Materials

The following supporting information can be downloaded at: https://www.mdpi.com/article/10.3390/brainsci13020185/s1, Table S1: Power analysis was performed in G*Power Statistical Power Analyses program for Mac and Windows, with the post hoc type of power analysis computed achieving power with a given α (0.05), sample size (*N* = 224), and effect size.
