# Supplementary material for: The Associations between Results in Different Domains of Cognitive and Psychomotor Abilities Measured in Medical Students
Source: Brain Sci. 2023 Jan 22;13(2):185. doi: 10.3390/brainsci13020185 (PMC9954177; doi:10.3390/brainsci13020185)
Supplement: Supplementary file 1 [file brainsci-13-00185-s001.zip › brainsci-2099855-supplementary.pdf]

Supplementary Table S1. Power analysis was performed in G*Power Statistical Power Analyses program for Mac and Windows, with the post hoc type of power analysis computed achieving power with a given α (0.05), sample size ($N = 224$), and effect size.

**Variable**		**APM**
**TTST 11**		
	Pearson's r	-0.480
	p-value	< .001
	Upper 95% CI	-0.370
	Lower 95% CI	-0.577
	Power (1-β)	1
**MINT 11**		
	Pearson's r	-0.436
	p-value	< .001
	Upper 95% CI	-0.320
	Lower 95% CI	-0.538
	Power (1-β)	0.999
**TTST 311**		
	Pearson's r	-0.296
	p-value	< .001
	Upper 95% CI	-0.169
	Lower 95% CI	-0.414
	Power (1-β)	0.998
**MINT 311**		
	Pearson's r	-0.330
	p-value	< .001
	Upper 95% CI	-0.206
	Lower 95% CI	-0.444
	Power (1-β)	0.999
**TTST 411**		
	Pearson's r	-0.440
	p-value	< .001
	Upper 95% CI	-0.326
	Lower 95% CI	-0.542
	Power (1-β)	1
**MINT 411**		
	Pearson's r	-0.303
	p-value	< .001
	Upper 95% CI	-0.177
	Lower 95% CI	-0.420
	Power (1-β)	0.999
